# Supplementary material for: What facilitates or prevents academic fraud in a Colombian faculty of medicine–Protocol of a study using fuzzy cognitive mapping
Source: PLoS One. 2023 Sep 20;18(9):e0291737. doi: 10.1371/journal.pone.0291737 (PMC10511091; doi:10.1371/journal.pone.0291737)

**Supplementary material 1. Advertisement to be used for the recruitment of participants.**

*Image with the invitation*


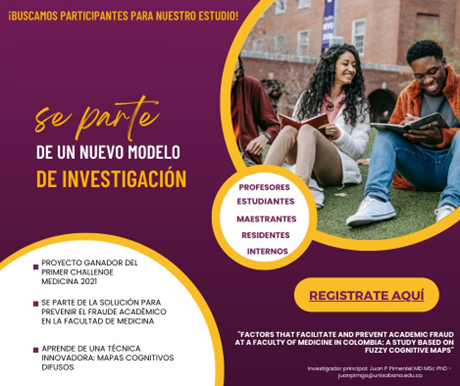


*WhatsApp message*


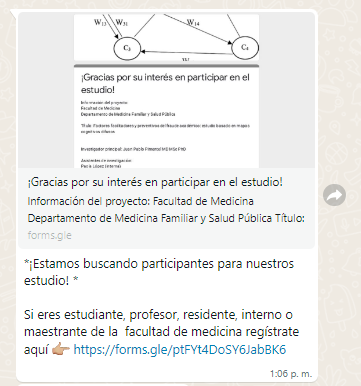

Supplement: S1 File — (DOCX) [file pone.0291737.s001.docx]
